# Assessing long-term, vestibulotoxic side effects after gentamicin therapy in neonatal sepsis or infection using video head impulse test

**DOI:** 10.3389/fped.2024.1366074

**Published:** 2024-02-26

**Authors:** Lena Zaubitzer, Anja Kotzur, Stefan Hegemann, Nicole Rotter, Angela Schell

**Affiliations:** ^1^ Department of Otorhinolaryngology, Head and Neck Surgery, University Hospital Mannheim, Medical Faculty Mannheim, University of Heidelberg, Mannheim, Germany; ^2^ Balance Clinic Zurich, Zurich, Switzerland

**Keywords:** gentamicin, vestibulotoxicity, newborn, sepsis, children, video head impulse test, gain, refixational saccades

## Abstract

**Study background:**

Newborn infection and sepsis remain serious problems. Guideline-compliant therapy includes, among other therapeutics, calculated intravenous antibiosis with gentamicin. One of the known side effects of gentamicin is severe vestibulotoxicity, which can be detected using the video head impulse test (VHIT), which is a sensitive examination method for the detection of vestibular hypofunction in children and adults. Previous studies on the vestibulotoxicity of gentamicin in newborns were carried out using caloric testing, rotary testing, and electronystagmography. Nevertheless, there are currently no data available on VHIT examinations in children who have been treated with neonatal gentamicin therapy.

**Methods:**

A single-center, prospective cross-sectional study, was conducted at a tertial referral center. VHIT was performed on 23 children aged 3–7 years who had received intravenous gentamicin therapy for at least five days as part of the treatment of newborn sepsis between 2012 and 2016. Main outcome was median gain and occurrence of refixational saccades as measured with VHIT. In addition, the children's parents received questionnaires to detect possible risk factors and vestibular and cochlear abnormalities.

**Results:**

Out of 23 children with a mean age of four years and seven months (ranging from 3 to 7 years), 11 (47.8%) indicated abnormal results in VHIT. The VHIT results were unilaterally abnormal in six children (26.1%) and bilaterally abnormal in five others (21.7%). Additionally, five of the children with an abnormal HIT had abnormalities, as found in the questionnaire results.

**Conclusion:**

and Relevance: Almost half of the children observed after having undergone gentamicin therapy as newborns showed abnormalities in VHIT, although they did not show any clinical signs of disbalance or vestibular hypofunction. VHIT can serve as a sensitive investigation method for the early screening of post-therapeutic vestibulotoxic side effects after gentamicin therapy in children. Additionally, VHIT can enable early intervention in these children.

## Introduction

Neonatal infections are a global problem, and neonatal sepsis is a major contributor to morbidity and mortality in newborns. In 2017, neonatal sepsis was identified as the third leading cause of newborn mortality (1). Guideline-compliant empirical therapy includes, among other therapeutics, a calculated systemic antibiotic therapy with aminoglycosides, especially gentamicin that is often administered as an empirical therapy in combination with ampicillin (1, 2). The known side effects of gentamicin include potential nephrotoxicity, cochleotoxicity and vestibulotoxicity (3, 4). A treatment duration of 5–7 days should be observed in newborns with positive infection parameters and uncomplicated clinical symptoms. If the pathogen is detected in the blood culture in clinical sepsis, the antibiotic treatment should be extended to 7–10 days (1). With regard to adverse drug reactions, short-term therapy of up to 5 days is preferable, as the risk increases significantly from a therapy duration of 8 days or if therapy is repeated within 6 weeks (5). Furthermore, unnecessary protracted antibiotic therapy should be avoided, as a higher mortality rate and an increased risk of necrotizing enterocolitis have been shown, especially in premature infants with a birth weight <1,000 g, with a treatment duration of ≥5 days (6).

The ototoxicity (both vestibular and/or cochlear) of aminoglycosides, including gentamicin, has already been well investigated in studies in adults (7). The toxicity of gentamicin appears to be greater for vestibular hair cells than for cochlear hair cells (8, 9). Despite the possible lasting effects of vestibulotoxicity on the development of children (10, 11), there are significantly fewer studies in children than in adults. The aim of the present study was therefore to gain new insights into the vestibulotoxicity of gentamicin in children.

Due to children's limited ability to communicate, vestibular dysfunction is often diagnosed late in this age group, which can affect their development (12, 13). Therefore, objective examination methods are indispensable for detecting vestibular dysfunction at an early stage so that affected children can be treated accordingly as soon as possible (14). Previous studies on the vestibulotoxicity of gentamicin in newborns were carried out using caloric testing, rotatory testing, and electronystagmography. The available studies did not show any significant results when using these methods (3, 15). Nevertheless, in adults, it has already been shown that the head impulse test (HIT) is a suitable method for detecting the early-stage vestibulotoxicity of gentamicin (16). In patients with bilateral vestibular loss, the head impulse test shows large overt-saccades and a gain reduction (17). In adults it is earlier and more often pathologic than hearing tests (18). We hypothesize that this is also the case in children, as VHIT in general is considered a suitable examination for the detection of vestibular functional impairment in children (11). Therefore, we sought to evaluate high-frequency vestibular ocular reflex (VOR) function in children who had undergone gentamicin therapy using VHIT.

## Materials and methods

In a single-center, prospective cross-sectional study conducted at the University Medical Center Mannheim, 23 children who had received systemic gentamicin therapy for at least five days between 2012 and 2016 as part of neonatal sepsis were examined.

During this four-year period, in total 235 children received intravenous systemic gentamicin therapy at the children's hospital. 163 of these 235 children (69.3%) received at least 5 days of gentamicin treatment and were therefore potentially eligible for this study.

Current contact information was available in 104 children of these eligible 163 children. The parents of those 104 children were contacted and finally 29 parents (27.8%) responded. Finally, 23 parents agreed to take part in the study and returned to the clinic for examination. The examination was performed at least three years after gentamicin therapy between 06/2018-01/2020. The parents of all study participants gave their informed consent to participate. The study was approved by the Ethics Committee II of the University of Heidelberg, Medical Faculty Mannheim (2018-503N-MA).

In all children included, neonatal sepsis was diagnosed via peripheral venous sampling of blood cultures with the preparation of an antibiogram and the laboratory chemical determination of CRP, IL-6 and/or IL-8 and a differential blood count. Gentamicin was administered intravenously as a one-hour infusion every 24 h at a dosage between 4.0 and 5.5 mg/kg depending on the gestational age. Trough levels were measured at least once during gentamicin treatment and were within target ranges in all children.

The VHIT was carried out using EyeSeeCam™ from Interacoustics A / S Denmark™ to register the patients’ VOR. The children's eye and head movements were measured with a high-speed infrared camera (250 images/second) with a built-in accelerometer. During the examination, the examiner stood behind the patient, who sat in front of a wall about 1.5 m away. The patient looked at a marked point on the wall at an angle of 20°. The system was then calibrated, and the children were instructed to focus on a goggle-emitted laser dot that was projected onto an animal motif on the wall. The patient's head is then moved in short, rapid movements without side prediction from the central position approximately 10°–20° to the right and left. Gain and standard deviation were automatically calculated by the EyeSeeCam™ software for the times 40 ms, 60 ms, and 80 ms. Two artifact-free head impulses per side are sufficient for an evaluable result, as published before (19). If saccades were found to be present, more than two head impulses were required. In addition, abnormalities in motor development (age of learning to walk, ride a bike) and the ear-nose and throat (ENT) medical history (headaches, migraines, ear diseases/operations, otitis, kinetosis) were asked for in a questionnaire.

If the child's newborn hearing screening data were not available or abnormal, otoacustic emissions (OAE) or brainstem evoked response audiometry (BERA) were performed to evaluate a potential hearing loss.

The outcome measures of the VHIT were median gain, standard deviation, and the presence of catch-up saccades. The standard values represent a mean gain of 1.02 ± 0.28. The presence of catch-up saccades or reduced gain (< 0.8) were rated as abnormal. The acquired mean gain values were compared with existing age-matched standard values using a *t*-test for independent samples (*p* ≤ 0.05), that were acquired during the same time at our clinic (11).

Based on the Denver Developmental Test (20), specific questions were asked about the development of gross and fine motor skills, language skills, and social contacts.

In addition, all study participants received an additional 10-item questionnaire for standardized assessment of possible pre-existing or concomitant vestibular disorders and developmental milestones. (Supplementary Material)

Furthermore, genetic syndromes or familiarity for hearing loss were ruled out by the family's medical history. Furthermore, a renewed ototoxic medication was ruled out.

Statistical analyses were carried out using SPSS Statistics Version 24.0 (SPSS Inc., Chicago, IL, USA).

## Results

The data of 23 children aged between three years and two months and a maximum of seven years (mean age = four years and seven months) were evaluated. The cohort consisted of 10 girls (43.48%) and 13 boys (56.52%). Two of the children were preterm (born before week 37). Mean duration of gentamicin therapy was 6.22 days. The family history revealed no evidence of a genetic defect or hereditary genesis of hearing loss in any of the children included. Furthermore, no child presented with hearing loss or had to undergo potentially ototoxic medication in the meantime. In detail, 5 children had abnormal newborn hearing tests that had already been carried out again by the time of study inclusion and were regular. Nevertheless, OAE measurement was performed in these five patients again, without any abnormalities. In one child, newborn hearing screening tests results were not available. OAE and BERA were preformed and did not show any pathologies. (Table 1 summarizes descriptive data).

**Table 1:** Descriptive data of all included children.

	Sex	Age	Prematurely born	Gentamicin (days)	Hearing test results	vHIT results	Motoric developtmental delay
1	F	5 years 2 months	No (37 w + 2 d)	7	Newborn HS normal	Abnormal	No
2	M	3 years 9 months	Yes (31 w + 4 d)	5	Newborn HS initially abnormal, OAEs repeatedly normal	Normal	No
3	F	6 years 6 months	No (37 w + 1 d)	7	Newborn HS normal	Abnormal	No
4	F	3 years 8 months	No (38 w + 6 d)	7	Newborn HS normal	Normal	No
5	M	3 years 10 months	No (39 w + 1 d)	5	Newborn HS normal	Abnormal	Able to walk ≥16 months,
6	M	5 years 0 month	No (38 w + 3 d)	7	Newborn HS normal	NORMAL	No
7	M	4 years 1 month	No (39 w + 4 d)	7	Newborn HS initially abnormal, OAEs repeatedly normal	normal	No
8	F	3 years 8 months	No (40 w + 2 d)	5	Newborn HS initially abnormal, OAEs repeatedly normal	Abnormal	No
9	M	3 years 8 months	No (36 w + 6 d)	5	Newborn HS normal	Normal	No
10	F	4 years 3 months	No (39 w + 2 d)	7	Newborn HS normal	Abnormal	No
11	F	3 years 6 months	No (40 w + 0 d)	7	Newborn HS normal	Abnormal	No
12	M	3 years 11 months	No (38 w + 1 d)	7	Newborn HS normal	Normal	Able to walk ≥16 months,
13	M	5 years 11 months	No (37 w + 6 d)	7	Newborn HS normal	Normal	Able to walk ≥16 months, delayed motoric developtment
14	F	3 years 2 months	No (38 w + 2 d)	5	Newborn HS initially abnormal, OAEs repeatedly normal	Normal	No
15	M	7 years 0 month	No (38 w 0 d)	5	Newborn HS initially abnormal, OAEs repeatedly normal	Normal	No
16	F	4 years 5 months	No (40 w + 5 d)	7	Newborn HS normal	Normal	No
17	M	5 years 7 months	No (39 w + 2 d)	5	Newborn HS normal	Abnormal	Able to walk ≥16 months, delayed motoric developtment
18	M	5 years 11 months	No (37 w + 6 d)	7	Newborn HS normal	Abnormal	Able to walk ≥16 months, delayed motoric developtment
19	F	3 years 7 months	No (37 w + 3 d)	6	Newborn HS normal	Normal	No
20	M	3 years 5 months	No (39 w + 5 d)	7	HS data missing; OAE and BERA normal	Abnormal	Able to walk ≥16 months,
21	F	5 years 11 months	No (38 w + 2 d)	7	Newborn HS normal	Abnormal	No
22	M	5 years 10 months	Yes (32 w + 6 d)	5	Newborn HS normal	Normal	No
23	M	6 years 8 months	No (38 w + 0 d)	6	Newborn HS normal	Abnormal	No

F, female; M, male; y, years; mo, month; w, weeks; d, days; gentamicin, duration of therapy in days.

HS, hearing screening; OAE: otoacoustic emissions.

The median horizontal gain on the right side ± standard deviation was 1.11 ± 0.23; on the left side, it was 0.99 ± 0.30. The *t*-test showed no significant difference from the normal value (*p* > 0.05).

In three out of 23 participants (13.0%), a left-sided gain reduction was found and in one participant (4.3%) a bilateral gain reduction was found. In none of these four participants did additional catch-up saccades occur. Two of those children had a conspicuous anamnesis as they had started walking ≥16 months.

One-sided overt saccades were found in three participants (13.0%). In four children (17.4%), overt saccades were found on both sides; in two of these cases, additional covert saccades were found on the right side. Three of these seven children (42.9%) had suspicious anamnesis.

A total of 15 (65.2%) participants had developmental delays, potential balance disorders, or risk factors for a vestibular disease in their medical history. Only five of these children also showed abnormalities in VHIT (Table 2 summarizes vHIT results and potential risk factors).

**Table 2:** vHIT results and potential risk factors median gain and standard deviation (SD) values of all 23 included children.

	vHIT Results		Potential risk factors developmental delay
	gain ± SD right	gain ± SD left	
1	1,31 ± 0,29	1,06 ± 0,41**^O^**	—
2	1,34 ± 0,66	1,33 ± 0,45	—
3	0,94 ± 0,19	0,95 ± 0,44**^O^**	Father: migraine
4	1,39 ± 0,49	1,20 ± 0,21	—
5	1,01 ± 0,15	0,46 ± 0,23**^R^**	Able to walk ≥16 months,
			Both parents migraine and kinetosis
6	0,95 ± 0,39	0,90 ± 0,34	Mother: migraine
7	0,86 ± 0,31	0,84 ± 0,42	Mother: migraine
8	0,69 ± 0,05**^R^**	0,64 ± 0,03**^R^**	Father: migraine
9	0,98 ± 0,14	1,11 ± 0,15	Child: kinetosis
10	0,94 ± 0,27	0,57 ± 0,20**^R^**	—
11	1,37 ± 0,20**^O^**	1,24 ± 0,42	Mother: migraine
			Child: kinetosis
12	1,06 ± 0,00	1,01 ± 0,63	Able to walk ≥16 months,
			mother: migraine
13	1,26 ± 0,12	0,96 ± 0,28	Able to walk ≥16 months,
			delayed motoric development
14	1,32 ± 0,28	1,01 ± 0,41	Child: kinetosis
15	1,10 ± 0,13	1,44 ± 0,39	—
16	1,04 ± 0,09	0,88 ± 0,40	—
17	1,40 ± 0,14**^O^**	1,12 ± 0,31**^O^**	Able to walk ≥16 months,
			kinetosis and delayed motoric development
18	1,53 ± 0,23**^O^**	1,04 ± 0,18**^O^**	Able to walk ≥16 months, delayed motoric development
19	1,01 ± 0,21	1,10 ± 0,21	Father: migraine
20	1,02 ± 0,06	0,75 ± 0,21**^R^**	Able to walk ≥16 months,
			both parents migraine, delayed motoric development
21	1,15 ± 0,19**^O,C^**	0,94 ± 0,12**^O^**	—
22	0,92 ± 0,52	1,05 ± 0,39	Episodic nystagmus,
			mother: migraine
23	1,00 ± 0,16**^O,C^**	1,11 ± 0,13**^O^**	—

Pathologic vHITs are marked. Occurrence of saccades are indicated by O respectively C, reduced gain by R.

## Discussion

The ototoxicity (both vestibular and/or cochlear) of aminoglycosides, including gentamicin, has already been well investigated in studies in adults (7). In contrast to nephrotoxicity, the vestibulotoxicity of gentamicin is irreversible (21). The toxicity of gentamicin appears to be greater for vestibular hair cells than for cochlear hair cells (8, 9). Despite the possible lasting effects of vestibulotoxicity on the development of children (10, 11), there are significantly fewer studies in children than in adults.

Most neonatal studies deal with the optimal dosage to reduce the nephrotoxic effects of gentamicin. It could be shown that the nephrotoxicity is reduced by longer dosing intervals (22, 23). German guidelines for bacterial infection in newborns recommend a single 4–5 mg dose of gentamicin per kilogram of body weight every 24–48 h, depending on gestational age and postnatal age (24). In our study, VHIT showed minor abnormalities in 11 out of 23 children (47.8%), which indicates that the guideline-based dosage was sufficiently low to avoid vestibulotoxic side effects.

Aust et al. also found no significant differences in rotatory tests with regard to nystagmus and spontaneous eye movements between children who received gentamicin as newborns and the control group. Therefore, they concluded that gentamicin at controlled therapeutic doses in neonates has only mild vestibulotoxic side effects (25).

Studies examining the cochleotoxicity of gentamicin in newborns did not demonstrate an increased rate of abnormal hearing screenings at a median maximum daily dose of 4.0 mg/kg/day, a median daily average dose of 3.8 mg/kg/day, a median cumulative dose of 12.1 mg/kg, and a median therapy duration of three days. However, it has also been suspected that high doses and long durations of gentamicin therapy may be associated with an increased risk of hearing damage (26, 27). To establish serum level limits and determine the optimal dosage of gentamicin in neonates, vestibulotoxicity may be more important to avoid than cochleotoxicity, as all children showed normal hearing results. Additionally, vestibular hair cells are more prone to gentamicin toxicity and HIT might be easier or at least as easy to perform in neonates than hearing tests.

Both the results of the study mentioned above and those of the present study indicate that a child's vestibular organ could be more resistant or capable of regeneration than the vestibular organs of adults. Other studies have already shown that, in contrast to cochlear hair cells, neonatal and adult vestibular hair cells have the ability to regenerate (28, 29). Regeneration in the adult sensory epithelium is limited and occurs primarily through the replacement of type II-like hair cells (30). The neonatal sensory epithelium, on the other hand, appears to be more robust, and new cells develop characteristics of hair cells type I and II (31). If one assumes that these research results can be transferred to the human sensory epithelium, this could mean that the human vestibular organ is more capable of regeneration in newborns than in adults.

The VOR response only reaches normal functioning within the first two months of life (32) by linking the visual signal paths (33), among other things. On the molecular and electrophysiological levels, some maturation processes of vestibular hair cells take place within their first few weeks of life (34). An investigation of the vestibular hair cells of mice showed that certain ion channels, which are also involved in gentamicin uptake, mature postnatally. With the development of these ion channels, the uptake of gentamicin into the cell, and thus its toxic effect, also increases (35). Thus, the vestibulotoxic effect of gentamicin may be lower in neonates.

In our study, no significant differences in VOR gain were found compared to the control group. However, seven participants showed unilateral or bilateral saccades, which were rated as conspicuous. Three of these seven children also had a conspicuous questionnaire with regard to vestibular symptoms. Since saccades are unphysiological in children (11, 32), this suggests gentamicin-induced vestibulotoxicity, especially in conjunction with the conspicuous questionnaire.

Although the study results by Aust et al. (25) reject the notion of vestibulotoxicity, this cannot be completely ruled out since rotary chair testing was used as the examination method. Rotary chair testing only examines the middle-frequency range of VOR, whereas VHIT examines the high-frequency range (36). The toxicity of gentamicin results in changes of high-frequency horizontal VOR (37); thus, compensatory reactions, such as saccades caused by gentamicin, might not have been detected by rotary chair testing.

Six study participants showed unilaterally pathological examination results (catch-up saccades or gain reduction). In the case of systemic therapy with gentamicin, bilateral damage is expected. In addition to bilateral functional losses (4, 38), unilateral functional losses were also observed after systemic gentamicin therapy (17, 39).

Another reason for unilaterally pathological examination results could be the increased examination duration, which causes children to lose concentration. This leads to only one-sided eye tracking and artifacts. Nevertheless, it is unexpected to see more than 50% with a unilateral damage. Unilateral recovery would also be a hypothetic explanation without evidence-based support.

In summary, we did not find a significantly reduced VOR function measured by VHIT in children who had undergone systemic gentamicin therapy. Nevertheless, to our knowledge, for the first time, we were able to identify refixational saccades as a indicator for altered VOR-function in children who received postnatal systemic antibiotic therapy with gentamicin. Accordingly, vHIT seems to be an appropriate diagnostic tool for vestibular screening in children undergoing systemic gentamicin therapy.

Nevertheless, our study has limitations that need to be mentioned: Our number of included children is relatively small. In addition, a selection bias cannot be ruled out, as parents who might have noticed a potential delayed motor development are presumably more likely to accept a study offer for further clarification than parents of children without any problems. Therefore, future studies with larger case numbers are needed to further investigate these findings. Ideally, baseline testing prior to gentamicin therapy and longitudinal testing would certainly be preferable.

However, baseline vHIT-testing in particular does not appear to be easily practicable, as antibiotic therapy takes place directly postnatally and vHIT testing results are only reproducible from the moment of visual fixation (11). However, repeated longitudinal vHIT-testing may lead to a better understanding of semicircular canal function after perinatal gentamicin administration. Vestibular evoked myogenic potentials can be considered as a possible alternative for assessing at least otolith function, as these can be derived from birth and data are even available for premature births (40). However, vHIT-testing should be performed at the earliest possible point in time to detect and to objectify possible vestibulotoxic damage at an early stage. Future studies should therefore also focus on the other semicircular canals as well as the otolith function to examine the entire vestibular function, especially, since macular hair cells seem to be more likely to be lesioned by gentamicin.

Meanwhile, it seems appropriate to propose a vestibular screening in children who had undergone gentamicin therapy for the early detection of potential vestibular hypofunction and to promptly initiate further diagnostics or treatment.

## Conclusion

Guideline-oriented intravenous gentamicin therapy in children with neonatal sepsis does not seem to lead to clinically significant vestibular hypofunction or delayed motoric development in children. Nevertheless, VHIT can serve as a sensitive investigation method for the early screening of post-therapeutic vestibulotoxic side effects after gentamicin therapy in children. Additionally, VHIT can enable early intervention for these children.

## Data Availability

The raw data supporting the conclusions of this article will be made available by the authors, without undue reservation.
