# Supplementary material for: Assessing long-term, vestibulotoxic side effects after gentamicin therapy in neonatal sepsis or infection using video head impulse test
Source: Front Pediatr. 2024 Feb 26;12:1366074. doi: 10.3389/fped.2024.1366074 (PMC10929264; doi:10.3389/fped.2024.1366074)
Supplement: Supplementary file 1 [file Datasheet1.docx]

1. At how many months old was your child able to walk freely?
   - 12-15 months
   - 16-20 months
   - 21-24 months
   - earlier/later: with ... months
2. At the age of how many years could your child ride a bike without training wheels?
   - 2-3 years
   - 4-5 years
   - 6-7 years
   - later with: ... years
   - My child rides with training wheels.
   - My child does not ride a bike.
3. Were there any abnormalities during the prenatal check-ups?

- yes
- no
- If yes, which ones?

1. Does your child often suffer from headaches?

- daily
- once a week
- two to three times a week
- more than three times a week
- once a month
- two to three times a month
- less often/never

1. Do you have a family history of migraines?

- Yes
- no
- If yes, who is affected?

1. Have you noticed any motor abnormalities in your child (e.g. unsteady walking, problems in sports lessons, etc.)?

- yes
- no
- If yes, which ones?

1. Does your child get nauseous when traveling in the car or on ships?

- Yes, in the car
- Yes, on the boat
- No

1. How often has your child had a middle ear infection?
   - not at all
   - once or twice
   - three to five times
   - more than five times
2. Has your child had or does your child have other ear diseases?

- Yes
- No
- If yes, which ones?

1. Has your child had ear surgery?
   - Yes
   - No
   - If yes: Which operation(s) was (were) performed?

Supplemental material: Questionnaire used for assessment of possible pre-existing or concomitant vestibular disorders and developmental milestones
